# Elastin Peptides as a Potential Disease Vector in the Pathogenesis of Pulmonary Emphysema: An Investigation of This Hypothesis

**DOI:** 10.3390/life15030356

**Published:** 2025-02-24

**Authors:** Jerome Cantor

**Affiliations:** School of Pharmacy and Allied Health Sciences, St. John’s University, 8000 Utopia Parkway, Queens, NY 11439, USA; cantorj@stjohns.edu

**Keywords:** elastic fibers, elastin peptides, pulmonary emphysema, desmosine, disease vector

## Abstract

The degradation of elastic fibers is a fundamental characteristic of pulmonary emphysema, resulting in the release of proinflammatory elastin peptides. The findings discussed in this paper support the hypothesis that these peptides act as carriers of disease, interacting with elastin receptor complexes that promote inflammation, elastic fiber damage, and airspace enlargement. Studies from our laboratory show that the breakdown of these fibers is significantly enhanced by intratracheal instillation of elastin peptides in a lipopolysaccharide-induced model of acute lung injury. This result is consistent with a mechanism of elastic fiber injury in which an expanding pool of elastin peptides generates further elastolysis. The accelerating release of the peptides results in a self-perpetuating disease process with the features of an epidemic, where self-replicating agents spread disease. As in the case of an epidemic, elastin peptides resemble disease vectors that transmit alveolar wall injury throughout the lung. This concept may provide a framework for developing novel therapeutic approaches specifically designed to protect elastic fibers from various enzymatic and oxidative insults, thereby slowing the progression of a disease with no robust treatment options.

## 1. Introduction

Elastic fibers play a critical role in the expansion and contraction of the lung during the respiratory cycle [1,2]. They support the architecture of alveolar walls, helping to maintain their shape during expansion and contraction. The degradation of these fibers leads to airflow obstruction, causing hyperinflation of the lungs and rupture of alveolar walls. As the elastic fiber network deteriorates, it may also stimulate an inflammatory response mediated by the release of elastin peptides from fragmented elastic fibers [3,4,5].

The proinflammatory effect of the peptides is mediated by their binding to the elastin receptor complex (ERC), which plays an important role in multiple biological processes ranging from tissue development and remodeling to cellular signaling pathways that govern cell migration and proliferation [6]. Structural studies using NMR spectroscopy and other techniques have provided insights into the conformational changes that occur upon binding, shedding light on the dynamics of the peptide–receptor interaction [7,8,9]. Elastin peptides with specific amino acid sequences, such as Val-Gly-Val-Ala-Pro-Gly, exhibit distinct bioactive properties and have been shown to stimulate the chemotaxis of immune cells, particularly neutrophils and macrophages [7,10]. This process amplifies the inflammatory response within the lung environment, increasing the production of elastin peptides and resulting in a self-perpetuating cycle of inflammation and tissue destruction.

Based on recent experimental findings, it is hypothesized that the advancement of emphysematous changes in the lung involves the dissemination of these peptides through an inflammatory cell population, resulting in the breakdown of elastic fibers, increased mechanical strain on alveolar walls, and airspace enlargement. As in the case of an epidemic, elastin peptides act as a vector for the spread of a self-replicating amino acid sequence responsible for disease propagation. This concept could provide a rationale for developing a treatment that inhibits the spread of these peptides in the lung, thereby slowing the progression of alveolar wall injury and reducing the risk of respiratory failure.

## 2. Role of Elastin in the Pathogenesis of Pulmonary Emphysema

### 2.1. Mechanical Properties of Elastic Fibers

The primary component of elastic fibers is the distensible elastin protein, which stores energy during lung expansion [11,12]. Elastin lacks the rigid, crystalline structure typical of other structural proteins like collagen, allowing it to stretch significantly without losing its structural integrity. This elasticity is primarily due to a unique amino acid composition rich in glycine, valine, and proline, enabling the protein to adopt flexible conformations. When elastin stretches during inhalation, the decrease in entropy is associated with energy storage. Conversely, returning to a more disordered state during exhalation releases the force needed to expel air from the lungs [13]. These changes in entropy are facilitated by the hydrophobic regions of elastin, which interact with neighboring water molecules [14].

### 2.2. Modeling the Role of Elastic Fiber Injury in Pulmonary Emphysema

The initial changes associated with the development of pulmonary emphysema involve only localized damage to elastic fibers. As the structural alterations in these fibers progress, they disrupt the normal distribution of mechanical forces, causing the rupture of alveolar walls [15]. This process may be modeled using percolation theory, which analyzes the movement of fluids through interconnected channels [16]. A particular percolation model, the random resistor network, may be utilized to investigate how changes in elastic fibers influence lung mechanics [17]. It involves the indiscriminate disconnection of conducting bonds, resulting in a decreased flow of electrical current. This process is analogous to the impaired transmission of mechanical forces through fragmented elastic fibers. It may be modeled by constructing a network composed of two interconnected units, K1 and K2, representing intact and disintegrating elastic fibers, respectively [18]. These components are randomly arranged within a three-dimensional lattice, and their relative proportions significantly impact mechanical force transmission throughout the lung.

**Figure 1:**
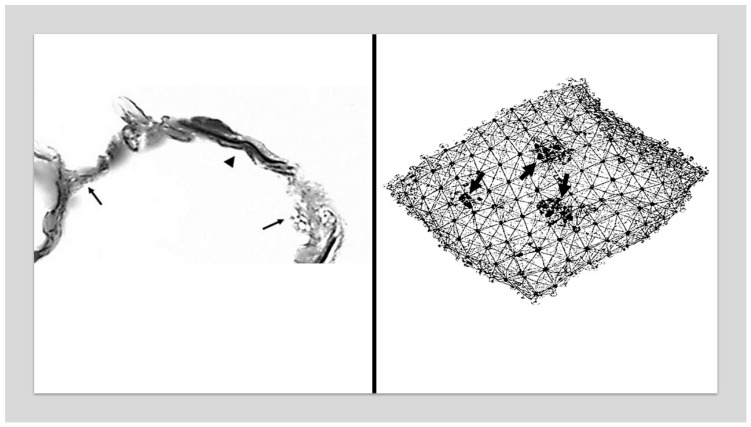
(**Left**) Photomicrograph of elastic fibers from a postmortem human emphysematous lung showing their unraveling (arrowhead) and fragmentation (arrows). Reprinted with permission [10]. (**Right**) Diagram of lung elastic fiber network showing intact (solid lines) and fragmented (dotted lines) fibers. Foci of alveolar wall distention and rupture (arrows) associated with the loss of intact fibers gradually undergo expansion and become confluent. Elastin peptides (individual dots) bind to elastin receptor complexes in the extracellular matrix and act as a vector in propagating emphysematous changes.

When the proportion of K2 units is low, the forces are evenly distributed across the K1 units, causing minimal changes in lung structure. However, as the number of K2 units increases due to elastolysis, the active transmission of mechanical forces in the lung becomes concentrated in the remaining K1 units. The additional strain on the K1 units promotes their transformation to K2 units, resulting in diminished elastic recoil, hyperinflation of the lung, and the rupture of alveolar walls (Figure 1).

### 2.3. Role of Elastin Peptides in the Pathogenesis of Pulmonary Emphysema

Early investigations conducted in the 1970s began to elucidate the connection between elastin degradation and the pathophysiology of emphysema. It was shown that proteolytic enzymes released by neutrophils and macrophages play a critical role in alveolar wall injury, laying the groundwork for subsequent research efforts focused on understanding the complex interactions between inflammatory responses and the structural integrity of the lung [19,20,21,22]. Elastolytic enzymes produced by these cells, including matrix metalloproteinases (MMPs) and serine proteases, are central to the pathogenesis of pulmonary emphysema [23,24].

These enzymes are synthesized in response to lung irritants like tobacco smoke and result in the generation of elastin peptide fragments that are critical mediators of inflammation in pulmonary emphysema. These peptides have been shown to interact with the ERC and activate immune responses [25]. The binding of elastin peptides to their receptors activates various intracellular signaling pathways, causing the upregulation of proinflammatory cytokines, further exacerbating lung damage [26].

Beyond attracting inflammatory cells, elastin peptides play an important role in lung remodeling by facilitating cell proliferation and extracellular matrix remodeling. By promoting the activation of fibroblasts and the secretion of elastases and other enzymes, these peptides can produce changes in matrix composition that adversely affect the mechanical properties of alveolar walls and contribute to the pathogenesis of emphysema [3,27]. Studies showed that intratracheal administration of elastin peptides in mice can induce alveolar wall inflammation, significantly increasing alveolar diameter compared to controls [28]. Elastin peptide-treated mice also demonstrated increased recruitment of neutrophils and macrophages, causing further degradation of elastic fibers [29].

The proinflammatory activity of these peptides is associated with their binding to the elastin binding protein, a 67 kDa component of the ERC [30,31]. Before the emergence of pulmonary emphysema, the fragmentation and unraveling of elastic fibers may expose the core elastin protein to enzymatic and oxidative breakdown, facilitating the release of proinflammatory peptides [10].

To investigate this hypothesis, our laboratory developed a hamster model of pulmonary emphysema using elastase and LPS to examine the relationship between lung inflammation and damage to elastic fibers [10]. The combination of these agents was informed by prior research showing their synergistic interaction in promoting airspace enlargement [32]. To enhance the impact of LPS, a single low dose of elastase was given to the hamsters with a shorter interval between the enzyme instillation and LPS administration, thereby improving the detection of potential synergistic effects. In contrast to earlier studies that employed multiple elastase treatments over several weeks before administering LPS, this revised model concentrated on a single dose and minimized the timeframe between the two treatments [33].

This model was used to assess whether pretreatment with elastase altered the structure of elastic fibers, making them more vulnerable to subsequent injury from LPS. The findings revealed that hamsters treated with elastase and LPS exhibited significantly higher levels of cells in bronchoalveolar lavage fluid (BALF) compared to those treated with elastase and saline, saline and LPS, or the control group. Furthermore, the proportion of neutrophils in the BALF was significantly greater in the animals receiving both elastase and LPS, in contrast to those treated with either elastase or LPS alone [10].

**Figure 2:**
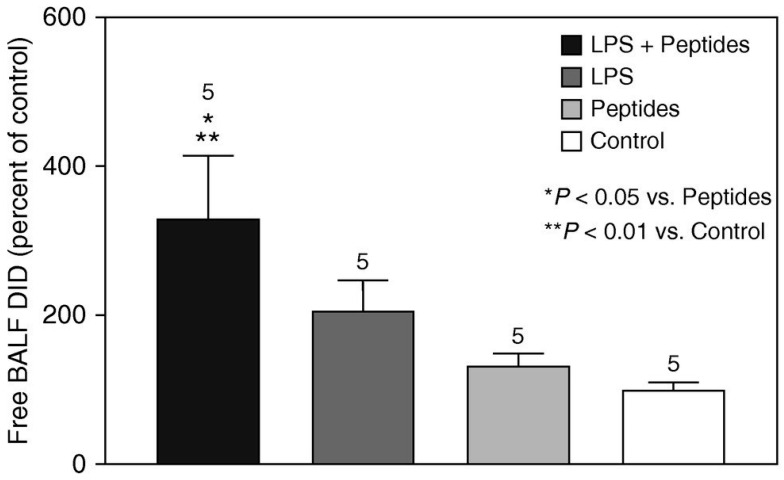
Concurrent intratracheal instillation of LPS and elastin peptides resulted in a significant increase in elastolysis as measured by BALF levels of free desmosine. The proinflammatory effect of the peptides is consistent with their role as a vector in the spread of airspace enlargement through the lungs. Reprinted with permission [10]. T-bars indicate the standard error of the mean (SEM). The numbers above bars denote N.

The proinflammatory effects of elastin peptides released from damaged elastic fibers were investigated in a lung injury model induced by LPS. When elastin peptides and LPS were administered together via intratracheal instillation, there was a marked increase in the levels of neutrophils and markers of inflammation in bronchoalveolar lavage fluid (BALF) compared to the administration of either agent on its own [10]. The proinflammatory effect of the peptides was also reflected by an increase in elastolysis, as measured by BALF desmosine levels (Figure 2).

**Figure 3:**
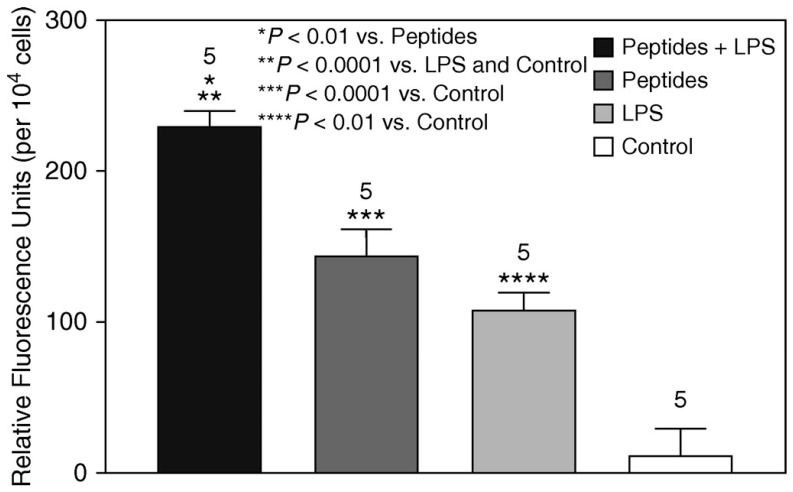
The combination of elastin peptides and LPS significantly increased the chemotactic activity of alveolar macrophages compared to either agent alone. Reprinted with permission [10]. T-bars indicate SEM. The numbers above bars denote N.

In vitro experiments were conducted using BALF macrophages from untreated animals to determine the chemotactic properties of elastin peptides [10]. While exposure to elastin peptides or LPS alone significantly increased chemotaxis compared to the control group, combining the two agents produced an even more significant enhancement in chemotactic response (Figure 3).

## 3. Elastin Peptides as a Vector in the Transmission of Alveolar Wall Injury

### Experimental Evidence for an Epidemic-like Mechanism in Pulmonary Emphysema

The relationship between excessive protease activity and damage to the alveolar wall is an important feature of pulmonary emphysema. However, other mechanisms may be more directly implicated in the development of the disease. Alterations in the distribution of mechanical forces could play a pivotal role in transforming proteolytic injury into airspace enlargement [34,35]. This concept is supported by in silico studies demonstrating that localized variations in alveolar wall elasticity can coalesce into widespread morphological changes mimicking those observed in pulmonary emphysema [36].

This observation is consistent with the principle of emergence, where complex interactions across multiple levels of scale lead to spontaneous reorganization of chemical and physical systems [37,38]. One example of an emergent phenomenon is an epidemic, which involves the unpredictable interplay of factors such as population density and mobility [39,40]. The incidence of infection tends to be uncertain until it reaches a critical threshold.

Similarly, the progression of pulmonary emphysema may incorporate comparable mechanisms in which indeterminate events govern the transition to an active disease state. Individual factors like elastase activity or antiprotease levels may only have a limited role in determining the extent of lung remodeling, which may instead require the identification of specific patterns of molecular and macroscopic behavior that reflect a self-organizing process operating across multiple scales [41].

**Figure 4:**
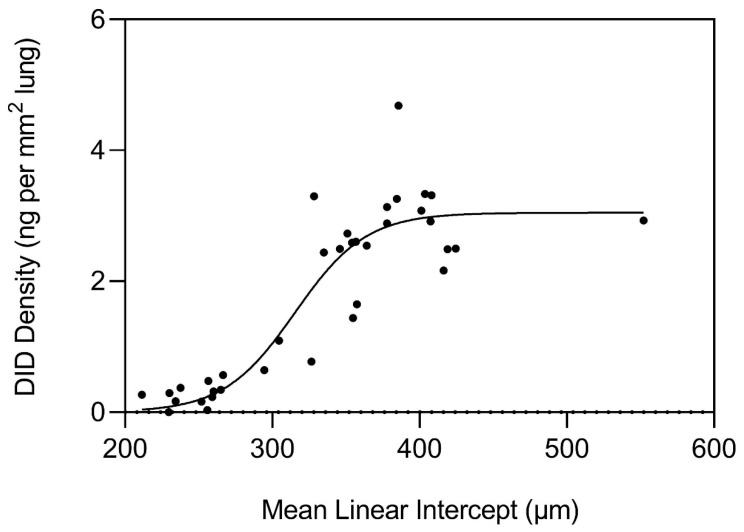
The repair of elastic fibers results in an exponential increase in desmosine crosslink density when the alveolar diameter exceeds 300 µm and levels off at 400 µm. Reprinted with permission [42].

**Figure 5:**
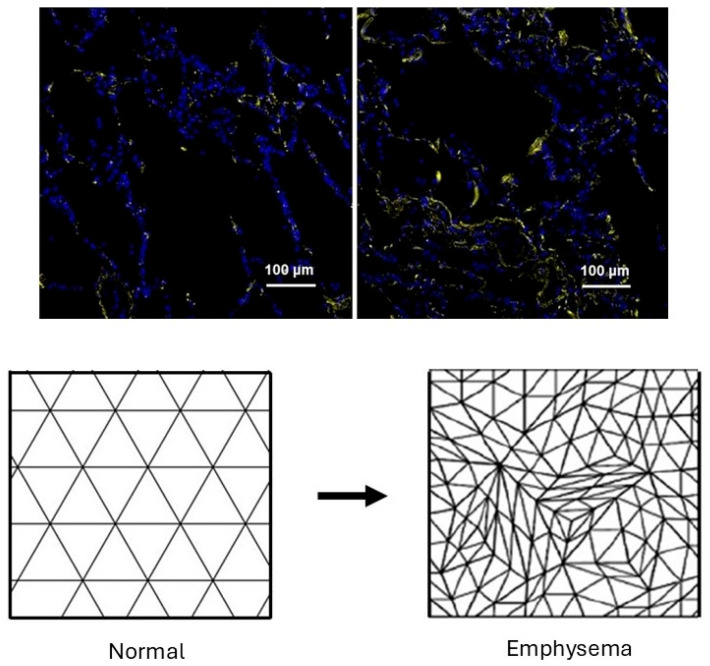
(**Upper**) Immunofluorescent staining for elastin shows increased deposition of this component in postmortem lungs with moderate pulmonary emphysema (**right**) compared to one with no disease (**left**). Reprinted with permission [42]. (**Lower**) Diagram modeling this process as a transition from an orderly network of elastin peptides (lines) and crosslinks (intersection points) to an irregular meshwork that produces uneven transmission of mechanical forces.

Measurements of elastin crosslinks in postmortem lungs from COPD patients provide evidence that an epidemic-like mechanism is responsible for elastic fiber injury and repair in pulmonary emphysema [42]. The main parameter used to evaluate “infection” is structural alterations in elastic fibers that result in the dysfunctional transmission of mechanical forces that induce airspace distention. As shown in Figure 4, desmosine crosslink density in lung tissue sections exponentially increases when the alveolar diameter exceeds 300 µm and plateaus beyond 400 µm. This finding is consistent with a repair process that initially counteracts alveolar wall injury and subsequently undergoes a decompensatory phase involving more widespread transmission of uneven mechanical forces that induce alveolar wall rupture. The increase in crosslinking is also accompanied by abnormal elastin deposition that further impairs energy storage by elastic fibers (Figure 5).

**Figure 6:**
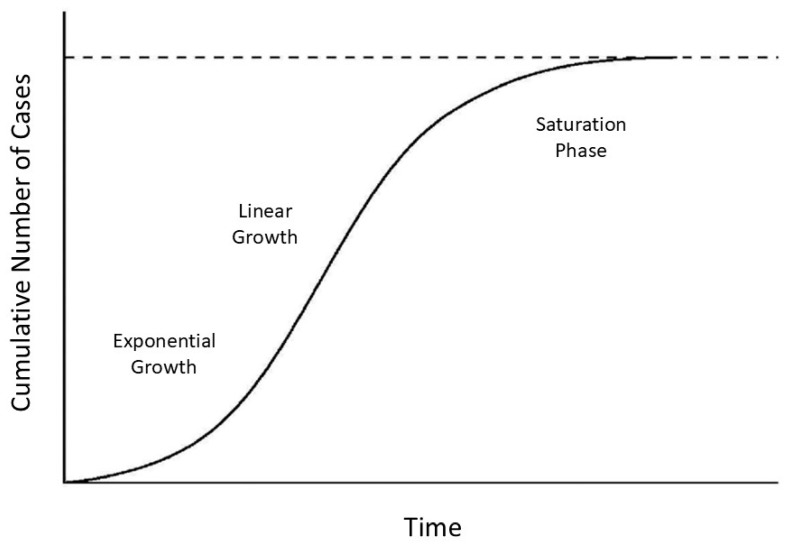
The curve of a communicable disease is similar to that associated with the increase in desmosine crosslink density (Figure 4). The dashed line indicates the upper limit of cases.

The shape of the crosslink density curve is similar to that of a communicable infection, where the cumulative number of cases is characterized by exponential, linear, and saturation phases (Figure 6) [43]. As in the case of an epidemic, the increase in dysfunctional elastic fibers results in a phase transition to an organized disease state reflected at increasing levels of scale by molecular changes in the extracellular matrix, microscopic airspace enlargement, and a loss of physiological function.

It is hypothesized that the spread of airspace enlargement through the lung requires a morphological landscape composed of a diffuse population of inflammatory cells subject to activation by the spread of elastin peptides that act as a vector for the progression of alveolar wall injury. This mechanism involves increasing elastic fiber injury that accelerates the release of elastin peptides, resulting in a self-perpetuating disease mechanism that contains the features of an epidemic.

An important feature of this process is its potential synergistic interaction with the uneven mechanical forces responsible for airspace distention and rupture [34]. The combination of elastin peptide-induced elastic fiber injury and loss of elastic recoil results in the progressive mechanical failure of alveolar walls and the further development of airspace enlargement. The continued spread of emphysematous changes eventually undergoes a phase transition to an active disease state involving a reorganization of lung architecture less amenable to therapeutic intervention.

## 4. Therapeutic Considerations

### 4.1. Leveraging the Relationship Between Pulmonary Emphysema and Epidemics

Similarly to physical systems, epidemics can undergo phase transitions where small changes in parameters, such as contact rates, can lead to abrupt changes in the outbreak dynamics, from endemic to epidemic or extinction [39]. Spatial dynamics also influence epidemic spread. The movement of people in geographic spaces leads to random spread and the formation of “hot spots” where the incidence of the disease is particularly high. Models incorporating spatial dynamics often exhibit complex emergent patterns arising from simple local rules about movement and transmission [44,45].

Both elastin peptide-induced lung injury and infectious epidemics are characterized by similar phases: initiation, exponential growth, stabilization, and resolution [43]. Initial exposure leads to localized responses that can escalate into widespread damage. Initially protective, an overactive inflammatory response results in tissue damage in both lung injuries and uncontrolled infections. Understanding these similarities could lead to preventive strategies, promoting therapeutic intervention before a phase transition involving irreversible lung injury.

### 4.2. Aerosolized Hyaluronan: A Potential Treatment for the Emergent Properties of Pulmonary Emphysema

The significant role of elastin-derived peptides in the pathogenesis of pulmonary emphysema suggests that reducing their levels in the lungs may slow the progression of the disease. While current therapeutic approaches have predominantly focused on elastase inhibitors for the treatment of pulmonary emphysema, our laboratory has pioneered the investigation of an aerosolized formulation utilizing low-molecular-weight hyaluronan (HA), a long-chain polysaccharide.

**Figure 7:**
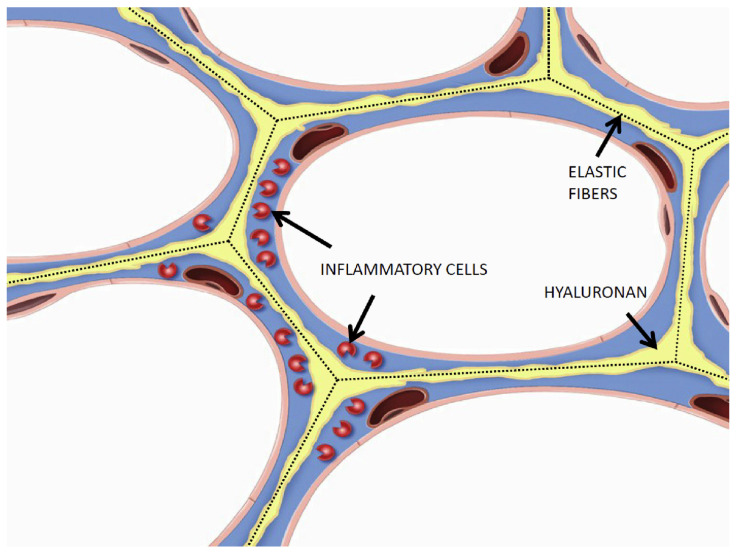
Aerosolized HA binds to elastic fibers, protecting them from injury and reducing the interaction of elastin peptides with inflammatory cells. Reprinted with permission from Elsevier.

Previous studies have shown that intratracheal instillation of hyaluronidase prior to induction of pulmonary emphysema with elastase significantly increased airspace enlargement, whereas instillation of hyaluronan (HA) in either elastase or cigarette smoke models of the disease had the opposite effect [46,47]. This protective effect is attributable to HA’s capacity to bind to elastic fibers, providing a physical barrier against agents that degrade elastin (Figure 7). The therapeutic potential of supplementing the extracellular matrix with exogenously administered HA was further supported by a study showing significantly reduced levels of this polysaccharide in the lungs of patients with alpha-1 antiprotease deficiency pulmonary emphysema.

The clinical efficacy of inhaling aerosolized HA was evaluated in a 28-day trial involving patients suffering from pulmonary emphysema due to alpha-1 antiprotease deficiency [48]. The study used desmosine levels in plasma, urine, and sputum to determine if elastin degradation was reduced. The results demonstrated that inhaling this agent twice daily resulted in a significant decrease in peptide-free urinary desmosine throughout the duration of the trial. This finding provides further evidence that aerosolized HA can diminish the release of elastin peptides and potentially slow the progression of alveolar wall injury.

An additional therapeutic effect of HA may involve its hydrophilic properties, which could enhance energy storage within elastin and alleviate the mechanical strain that contributes to the fragmentation of elastic fibers. This concept is corroborated by research suggesting that HA and other proteoglycans play a crucial role in mitigating the uneven distribution of forces within the extracellular matrix [49].

The potential effects of HA on multiple mechanisms of lung injury emphasize the need to develop holistic therapeutic strategies focused on more than one inflammatory pathway. Other than the use of alpha-1 antiproteinase in a small subset of COPD patients, agents that block a single inflammatory component have shown limited efficacy [50,51,52,53]. This lack of success may stem from the intricate nature of emerging phenomena, where system reorganization is contingent upon numerous interactions across different levels of scale.

### 4.3. Targeting Convergence Points in the Pathogenesis of Pulmonary Emphysema

Since the release of elastin peptides reflects a number of pathogenetic mechanisms, it may represent a point in the emergence of pulmonary emphysema where a group of agents interact concurrently to form a converging whole. Blocking the effects of these peptides by using antibodies to prevent their attachment to elastin receptor complexes or chemically modifying these receptors or their elastin binding protein component could provide alternative approaches to therapeutic intervention.

Pharmacological agents that selectively inhibit signaling through the ERC or modulate its interaction with elastin are currently under investigation. Potential drug candidates include small molecules that target pathways associated with ERCs, monoclonal antibodies that disrupt elastin interactions with this receptor complex, and gene therapy approaches aimed at downregulating its expression or activity [30].

While preclinical data highlight the potential of targeting the ERC for treating pulmonary emphysema, challenges remain in translating these findings into clinical practice. Safety profiles, pharmacokinetics, and patient variability need careful evaluation through well-designed clinical trials. Identifying reliable biomarkers to predict response to ERC-targeting therapies will be crucial for patient stratification and optimizing treatment outcomes. Future studies should focus on developing biomarker panels associated with ERC signaling pathways.

Considering the complex nature of pulmonary emphysema, a multi-modal approach combining ERC inhibitors with other proposed treatments, such as aerosolized HA, may produce synergistic interactions that greatly enhance therapeutic efficacy. This strategy may be particularly suited to the complexity of emergent processes involved in the pathogenesis of this disease.

### 4.4. Potential Consequences of Inhibiting Elastin Peptide Activity

Despite our understanding of the proinflammatory role of elastin peptides, the potentially harmful effects of inhibiting their activity remain unclear. Elastin peptides have been shown to regulate the proliferation and migration of cells involved in lung repair processes, such as fibroblasts and epithelial cells [6,7]. and may prevent excessive fibrogenesis. Furthermore, the ability of elastin peptides to activate matrix metalloproteinases may limit the accumulation of extracellular matrix that contributes to uneven mechanical strain on alveolar walls [7]. Maintaining a more balanced distribution of mechanical forces may slow the progression of airspace enlargement and reduce the risk of respiratory failure [15].

These potentially useful processes suggest that treatment with HA and other agents to reduce the formation of elastin peptides may need careful monitoring to prevent adverse consequences associated with the remodeling of the alveolar wall extracellular matrix. Measurement of the trajectory of biomarkers such as DID may provide an important indicator of a transition to a more active disease state.

## 5. Conclusions

The studies discussed in the current paper support the role of elastin-derived peptides in the pathogenesis of pulmonary emphysema. These peptides interact with specific receptors, initiating signaling events promoting inflammation and elastic fiber damage. The resulting airspace enlargement correlates with an increase in elastin crosslink density, and the graph of that relationship corresponds to that associated with the transmission of a communicable disease.

While this finding suggests that elastin peptides act as a vector in propagating alveolar wall injury, further studies are warranted to validate this concept, including clinical trials that determine the therapeutic effects of inhibiting elastin peptide activity. This treatment approach could slow the progression of pulmonary emphysema and significantly lower the risk of respiratory failure.

## Data Availability

Data are contained within the article.
